# Assessing concordance between patient-reported and investigator-reported CTCAE after proton beam therapy for prostate cancer

**DOI:** 10.1016/j.ctro.2021.09.003

**Published:** 2021-09-15

**Authors:** Roman O. Kowalchuk, David Hillman, Thomas B. Daniels, Carlos E. Vargas, Jean-Claude M. Rwigema, William W. Wong, Bradley J. Stish, Amylou C. Dueck, Richard Choo

**Affiliations:** a Department of Radiation Oncology, Mayo Clinic, Rochester, MN, USA; b Department of Statistics, Mayo Clinic, Rochester, MN, USA; c Department of Radiation Oncology, NYU Langone Health, New York, NY, USA; d Department of Radiation Oncology, Mayo Clinic, Phoenix, AZ, USA; e Department of Statistics, Mayo Clinic, Phoenix, AZ, USA

**Keywords:** Prostate cancer, Proton beam therapy, Patient-reported outcomes, Toxicity

## Abstract

**Purpose:**

We report acute patient-reported outcomes using CTCAE (PRO-CTCAE) of proton beam radiotherapy for high-risk or unfavorable intermediate-risk prostate cancer in a prospective clinical trial. PRO-CTCAE were correlated with investigator reported-CTCAE (IR-CTCAE) to assess the degree of concordance.

**Methods and materials:**

11 PRO-CTCAE questions assessed gastrointestinal (GI), genitourinary (GU), or erectile function side effects. The correlation scheme between PRO-CTCAE and IR-CTCAE was independently developed by two physicians. Analyses of PRO-CTCAE and IR-CTCAE were conducted using both descriptive terms and the converted grade scores. The Kappa statistic described the degree of concordance.

**Results:**

55 patients were included. IR-CTCAE underestimated diarrhea compared to PRO-CTCAE at the end of treatment (EOT), with a 28% rate of underestimation (11% by ≥ 2 toxicity grades). Similarly, urinary tract pain was underestimated in 45% of cases (17% by ≥ 2 grades) at EOT. Differences were less pronounced at baseline or 3 months after radiotherapy. The incidence of urinary urgency and frequency tended to be overestimated prior to treatment (36% and 24%, respectively) but underestimated at EOT (35% and 31%, respectively). The degree of interference with daily activities was consistently overestimated by investigators (45%-85%). Finally, erectile dysfunction showed a 36–56% rate of discordance by ≥ 2 toxicity grades.

**Conclusions:**

Our study shows a low agreement between IR-CTCAE and PRO-CTCAE in the setting of proton therapy for prostate cancer. Compared to patient-reported outcomes, physicians underestimated the frequency and severity of urinary symptoms and diarrhea at the end of treatment. Continued use of PROs should be strongly encouraged.

## Highlights

• Acute patient-reported outcomes were compared with investigator-reported outcomes.
• We identified low agreement between IR-CTCAE and PRO-CTCAE for prostate cancer.
• Physicians underestimated the frequency and severity of GU symptoms and diarrhea.
• The end of treatment was a key timepoint showing high levels of discordance.

## Introduction

The benefits of using patient-reported outcomes (PROs) are well established, with increased importance being placed on the patient perspective [1]. There are significant variations in the use of PROs in clinical trials, and novel ways to validate PROs and implement them into clinical workflows are needed [2], [3], [4].

PROs have been utilized to assess the impact of radiotherapy on the quality of life for a variety of diseases, and they have helped to guide the selection of an appropriate dose-fractionation regimen in some clinical settings [5], [6], [7], [8]. For example, PROs have been used to identify dosimetric factors associated with decreased quality of life for head and neck cancer patients undergoing radiotherapy; however, it has been also recognized that PROs may demonstrate significant differences compared with those recorded by physicians [9], [10].

There is strong interest among clinicians to incorporate validated PROs into clinical practice for prostate cancer patients to characterize genitourinary (GU) and gastrointestinal (GI) side effects [11], [12]. In a phase III clinical study of 1643 men with clinically localized prostate cancer, the results of PROs demonstrated that radical prostatectomy (RP) was associated with a greater detriment to urinary continence and sexual function than radiotherapy therapy (RT). However, RT resulted in a greater detriment to urinary voiding and nocturia than RP or active surveillance at 6 months post-treatment, although these symptoms recovered and were similar to RP or active surveillance at 12 months. Bowel function was worse with RT at 6 months than RP or active surveillance, but then partially recovered at 12 months [13].

There are several instruments available to measure PROs in prostate cancer. For example, EPIC (Expanded Prostate Cancer Index Composite) is a widely utilized tool to assess patient-reported quality of life. Recently, Patient-Reported Outcomes Version of the Common Terminology Criteria for Adverse Events (PRO-CTCAE) has been developed as a novel, validated, and reliable method for reporting patient-reported adverse events [14], [15], [16], [17]. Because investigator-reported CTCAE (IR-CTCAE) often under-detects symptomatic adverse events, PRO-CTCAE can be used to enhance the precision and patient-centeredness of adverse event reporting in cancer clinical research [18], [19]. In this study, we report acute PRO-CTCAE of proton beam therapy for patients with high or unfavorable-intermediate risk prostate cancer who received radiotherapy to the prostate/seminal vesicles and the regional pelvic lymph nodes in a prospective clinical trial. In addition, the correlation of PRO-CTCAE with IR-CTCAE is evaluated for discordance.

## Methods and materials

### Patient cohort

A prospective study was designed to assess the outcomes of moderately hypofractionated proton radiotherapy for high-risk or unfavorable intermediate-risk prostate cancer. This trial was approved by our institutional review board, and registered at clinicaltrials.gov (NCT02874014). Target accrual of 56 patients was attained in December 2018. Details concerning the inclusion criteria, dose-fractionation regimen, CT simulation, volume guidelines, treatment planning, and radiotherapy delivery are described in the supplemental documentation. Of note, patients also received androgen-deprivation therapy (ADT) for 4–36 months as part of the treatment paradigm of high or unfavorable intermediate risk prostate cancer.

### Investigator-reported CTCAE (IR-CTCAE)

IR-CTCAE were collected using the National Cancer Institute Common Terminology Criteria for Adverse Events, version 4 (CTCAE v4.0). Adverse events were assessed at baseline (prior to RT), weekly during RT, at the end of RT, 3-, 6-, and 12-months post-RT, and every 6 months up to 60 months post-RT.

In the study, 7 GI categories of CTCAE v4.0 were used to assess GI adverse events: diarrhea, proctitis, fecal incontinence, rectal stenosis, rectal ulcer, rectal hemorrhage, and small intestinal obstruction. For the evaluation of GU adverse events, 9 GU categories were used: urinary frequency, urinary urgency, urinary tract obstruction, urinary tract pain, urinary incontinence, hematuria, non-infective cystitis, urinary retention, and bladder spasm. For the assessment of erectile dysfunction adverse event, erectile function category was used. Adverse event grades were assigned by an attending physician or a clinical assistant, who also administered the CTCAE questionnaire.

### Patient-Reported outcomes version of the CTCAE (PRO-CTCAE)

The PRO-CTCAE questions used in the study were selected as the most relevant to prostate cancer patients undergoing radiotherapy and androgen deprivation therapy. They included 7 questions in the GI domain, 7 questions in the GU domain, 10 questions in the endocrine and erectile dysfunction domains, and 1 concerning skin toxicity. The selection process of these questions was subjective and based on the expected side effects from RT and/or ADT. These PRO-CTCAE questions were administered at baseline, at the end of RT, 3-, 6-, and 12-month post-RT, and every 6 months up to 60 months post-RT.

### Correlating PRO-CTCAE with IR-CTCAE

Few attempts have been made to correlate PRO-CTCAE with IR-CTCAE, and the most robust effort involved a panel of clinical investigators developing a general correlation [20]. In our study, there were 11 PRO-CTCAE questions that were deemed very closely corresponding to the IR-CTCAE definitions of a GI, GU, or erectile function adverse event. This correlation between PRO-CTCAE and IR-CTCAE in our study was conducted by two physicians independently, and the results demonstrated negligible disagreement. Correlating PRO-CTCAE descriptive grades to IR-CTCAE grading scores was conducted for each category of GI, GU and erectile function domains. This effort involved equating the descriptive term of each PRO-CTCAE grade to that of the IR-CTCAE grading score to have the most appropriate comparableness between the two instruments. In IR-CTCAE, some categories do not have a grade score 3 or higher designation (e.g. urinary frequency and urgency). Therefore, in such instances, an IR-CTCAE grading score that could be assigned for the worst descriptive PRO-CTCAE was limited by the maximum grade score available in a given IR-CTCAE category.

The corresponding adverse events and grades between the PRO-CTCAE questions and the IR-CTCAE categories used in our study are depicted in Table 1. This novel correlative effort between patient-reported vs. investigator-reported CTCAE has not been previously validated; thus, the analyses of the concordance and discordance between PRO-CTCAE and IR-CTCAE were conducted using both descriptive terms, as well as the correlated grade scores, per Table 1.

**Table 1:** Patient-reported outcome questions assessed are tabulated. Patient responses were then correlated with the grades of investigator-reported outcomes, via CTCAE v4.0. A summary of the CTCAE grading definition is included in parentheses. ADL = activity of daily living.

PRO Question	IR-CTCAE correlate	Response/Assigned IR-CTCAE grade	Response/Assigned IR-CTCAE grade	Response/Assigned IR-CTCAE grade	Response/Assigned IR-CTCAE grade	Response/Assigned IR-CTCAE grade
In the last 7 days, how OFTEN did you have loose or watery stools?	Diarrhea	Never/grade 0	Rarely/grade 0	Occasionally/ grade 1 (increase of < 4 stools per day over baseline; mild increase in ostomy output compared to baseline)	Frequently/grade 2 (increase of 4–6 stools per day over baseline; moderate increase in ostomy output compared to baseline)	Almost constantly/grade 3 (increase of ≥ 7 stools per day over baseline; severe increase in ostomy output compared to baseline; limiting self care ADL)
In the last 7 days, how OFTEN did you lose control of bowel movements?	Fecal incontinence	Never/grade 0	Rarely/grade 0	Occasionally/ grade 1 (occasional use of pads required)	Frequently/grade 2 (daily use of pads required)	Almost constantly/grade 3 (severe symptoms; elective operative intervention indicated)
In the last 7 days, what was the SEVERITY of your pain in the abdomen (belly area) at its WORST?	Proctitis	None/grade 0	Mild/grade 1 (rectal discomfort, intervention not indicated)	Moderate/grade 2 (symptoms; medical intervention indicated; limiting instrumental ADL)	Severe/grade 3 (severe symptoms; fecal urgency or stool incontinence; limiting self care ADL)	Very severe/grade 4 (life-threatening consequences; urgent intervention indicated)
In the last 7 days, were there times when you had to urinate frequently?	Urinary frequency	Never/grade 0	Rarely/grade 0	Occasionally/ grade 1 (present)	Frequently/grade 2 (limiting instrumental ADL; medical management indicated)	Almost constantly/grade 2 (limiting instrumental ADL; medical management indicated)
In the last 7 days, how much did frequent urination INTERFERE with your usual or daily activities?	Urinary frequency	Not at all/ grade 0	A little bit/ grade 0	Somewhat/grade 1 (present)	Quite a bit/grade 2 (limiting instrumental ADL; medical management indicated)	Very much/grade 2 (limiting instrumental ADL; medical management indicated)
In the last 7 days, how OFTEN did you feel an urge to urinate all of a sudden?	Urinary urgency	Never/grade 0	Rarely/grade 0	Occasionally/ grade 1 (present)	Frequently/grade 2 (limiting instrumental ADL; medical management indicated)	Almost constantly/grade 2 (limiting instrumental ADL; medical management indicated)
In the last 7 days, how much did sudden urges to urinate INTERFERE with your usual or daily activities?	Urinary urgency	Not at all/ grade 0	A little bit/ grade 0	Somewhat/grade 1 (present)	Quite a bit/grade 2 (limiting instrumental ADL; medical management indicated)	Very much/ grade 2 (limiting instrumental ADL; medical management indicated)
In the last 7 days, how OFTEN did you have loss of control of urine (leakage)?	Urinary incontinence	Never/grade 0	Rarely/grade 0	Occasionally/grade 1 (occasional, pads not indicated)	Frequently/grade 2 (spontaneous; pads indicated; limiting instrumental ADL)	Almost constantly/grade 3 (intervention indicated; operative intervention indicated; limiting self care ADL)
In the last 7 days, how much did loss of control of urine (leakage) INTERFERE with your usual or daily activities?	Urinary incontinence	Not at all/grade 0	A little bit/grade 0	Somewhat/grade 1 (occasional, pads not indicated)	Quite a bit/grade 2 (spontaneous; pads indicated; limiting instrumental ADL)	Very much/grade 3 (intervention indicated; operative intervention indicated; limiting self care ADL)
In the last 7 days, what was the SEVERITY of your pain or burning with urination at its WORST?	Urinary tract pain	None/grade 0	Mild/grade 1 (mild pain)	Moderate/grade 2 (moderate pain; limiting instrumental ADL)	Severe/grade 3 (severe pain; limiting self care ADL)	Very severe/grade 3 (severe pain; limiting self care ADL)
In the last 7 days, what was the SEVERITY of your difficulty getting or keeping an erection at its WORST?*	Erectile dysfunction	None/grade 0	Mild/grade 1 (decrease in erectile function but intervention not indicated)	Moderate/grade 2 (decrease in erectile function, erectile intervention indicated)	Severe/grade 3 (decrease in erectile function but erectile intervention not helpful; placement of a permanent penile prosthesis indicated)	Very severe/grade 3 (decrease in erectile function but erectile intervention not helpful; placement of a permanent penile prosthesis indicated)

*Patients were also given the option to select “Not sexually active” or “Prefer not to answer.”

### Statistics

The Kappa statistic was utilized to describe the degree of correlation between PRO-CTCAE and IR-CTCAE. Values ≤ 0 indicate no agreement, with values of 0.01–0.20, 0.21–0.40, 0.41–0.60, 0.61–0.80, and 0.81–1.00 corresponding to none to slight, fair, moderate, substantial, and almost perfect agreement, respectively.

## Results

### Patient characteristics

Fifty-five patients completed radiotherapy, and baseline characteristics were described in Table 2. Median age was 75 years (range: 55–87). Most patients (95%) had high-risk prostate carcinoma. Median PSA was 10.24 ng/mL (range: 0.65–97.3).

**Table 2:** Patient characteristics are shown.

Characteristic	Incidence
Median age (years)	75 (55–87)
Median baseline PSA (ng/mL)	10.24 (0.65–97.3)
Median duration of ADT (months)	18 (4–37)
**Gleason score** 6 7 8–10	5 (9%) 17 (31%) 33 (60%)
**T stage** T1-T2 T3a T3b	23 (42%) 22 (40%) 10 (18%)
**Risk category** High-risk Unfavorable intermediate-risk	52 (95%) 3 (5%)

All patients were provided with the list of PRO-CTCAE questions. One patient routinely failed to complete these questions. For erectile dysfunction, many patients chose the “Prefer not to answer” or “Not sexually active” options, which made the correlation with IR-CTCAE v4.0 very difficult.

### IR-CTCAE

**Fig. 1:**
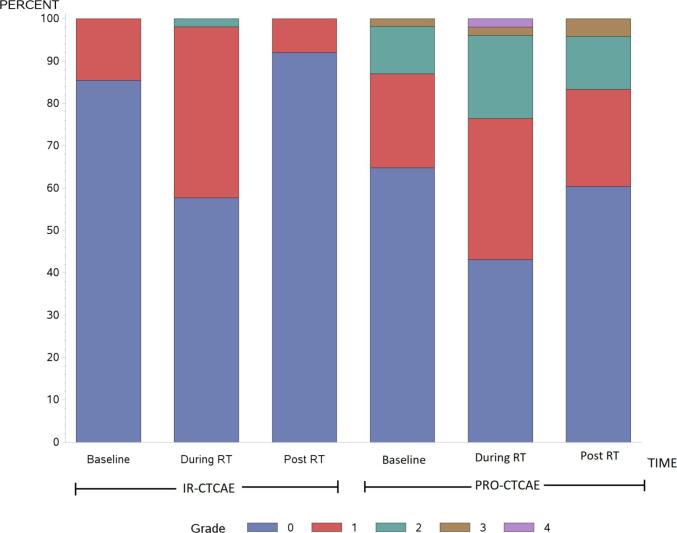

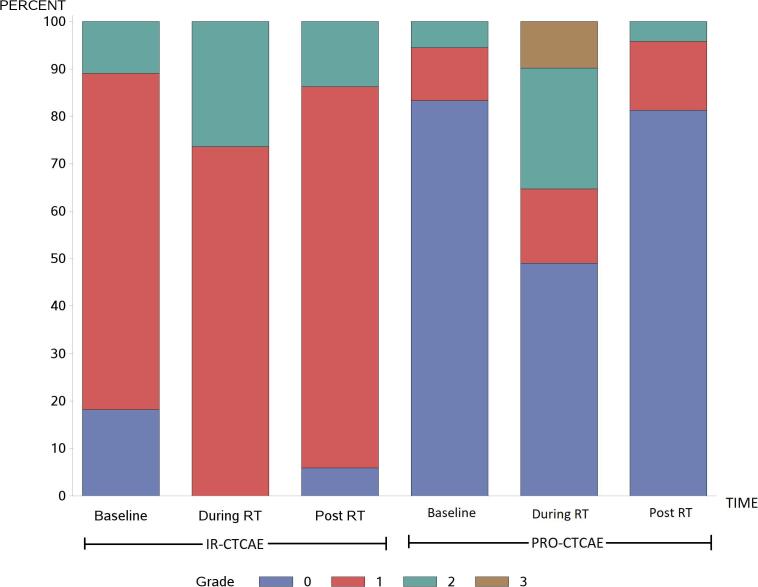
The rate of grade 0–4 toxicity is illustrated, as recorded via IR-CTCAE and PRO-CTCAE at baseline, during radiotherapy, and 3 months after radiotherapy. GI (a) and GU (b) toxicity rates are shown separately.

IR-CTCAE are demonstrated in Fig. 1a and 1b, for GI and GU toxicities, respectively. At baseline, most patients (94%) had no diarrhea. Fecal incontinence was similarly uncommon. A small portion of patients (9%) had baseline grade 1 proctitis. Urinary symptoms at baseline were more common: 74% with ≥ grade 1 urinary frequency and 49% with ≥ grade 1 urgency. Most patients (71%) had some degree of erectile dysfunction at baseline.

At the end of RT, IR-CTCAE registered an increase in diarrhea (31% with grade 1) but no other significant changes in GI adverse events. GU adverse events also increased at the end of RT, with most patients suffering from either grade 1 (70%) or grade 2 (28%) urinary frequency. Most patients (72%) had grade 1 urinary urgency. Urinary tract pain (≥grade 1) increased from 11% at baseline to 31% at the end of RT. Some degree of erectile dysfunction was registered in the majority of patients (79%).

Most GI and GU symptoms normalized 3 months after radiation therapy. Urinary frequency, however, remained increased from baseline (94% ≥ grade 1, and 15% grade 2). Erectile dysfunction also remained worse than baseline, with only 14% of patients with grade 0 and 46% with grade 2.

### PRO-CTCAE

**Fig. 2:**
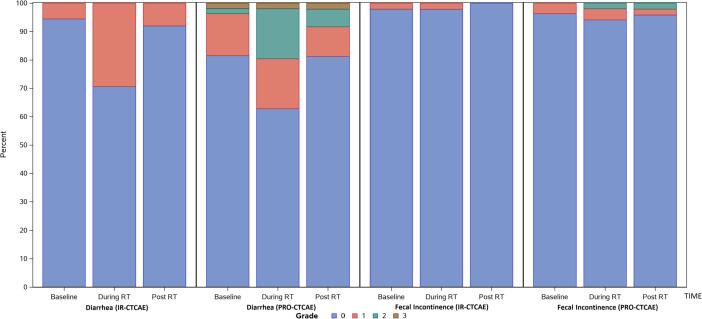
The rates and corresponding grades of diarrhea and fecal incontinence via IR-CTCAE and PRO-CTCAE are shown at baseline, during radiotherapy, and 3 months after radiotherapy.

PRO-CTCAE were assessed in their raw, non-converted forms, as well as with the correlations to IR-CTCAE v4.0 outlined in Table 1. Raw PRO-CTCAE revealed increased “frequent” diarrhea at the end of treatment (18%) versus baseline (2%) (Fig. 1a). The converted values similarly reported an increase in grade 2 diarrhea at the end of treatment (18%) versus baseline (2%). Proctitis showed an increase in “mild” pain at the end of treatment (34%) versus baseline (13%), which directly mirrored the converted results. GI symptoms recovered 3 months after treatment. Fecal incontinence was uncommon (Fig. 2).

**Fig. 3:**
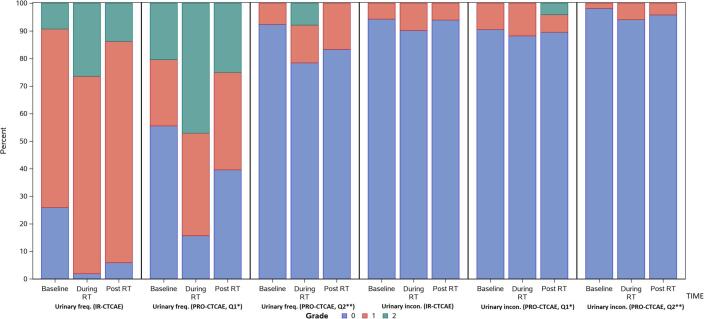

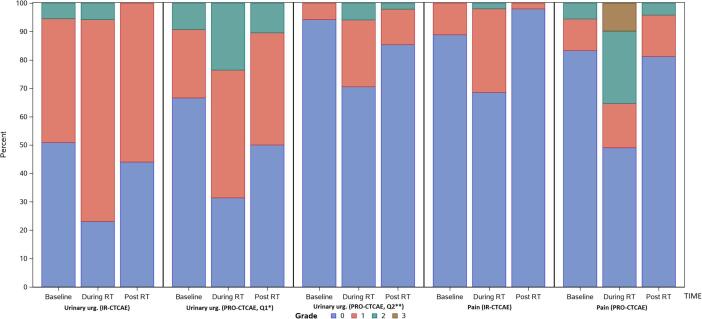
A comparison of the rates and grades of urinary frequency and urinary incontinence are shown, with Q2 referring to the interference of the symptom with daily activities and Q1 referring to the frequency of the symptom (a). Urinary urgency and urinary tract pain are similarly portrayed (b).

Urinary frequency and urgency were addressed by two different PRO-CTCAE questions. First, frequency was queried (ranging from “never” to “almost constantly”). In the non-converted form, increases were noted in urinary frequency “occasionally,” frequently,” and “almost constantly” at the end of treatment, with rates of 36% vs. 24%, 38% vs. 20%, and 10% vs. 0%, compared to baseline, respectively. Mild increases in urinary urgency were also common at the end of treatment (converted grade 2: 24% vs. 9% at baseline). Both frequency and urgency improved three months after radiotherapy, but there remained a mild increase in urinary frequency “occasionally,” compared to baseline (37% vs. 24%). Using the converted values, grade 2 urinary frequency showed a similar pattern: it increased to 48% at the end of treatment (vs. 20% at baseline). It subsequently declined, but it remained slightly elevated (24% 3 months after radiotherapy) (Fig. 3).

When patients were asked about how urinary symptoms “interfered” with “usual or daily activities,” much lower rates of adverse events were reported. Only 8% and 6% of patients reported that urinary frequency and urgency, respectively, interfered “quite a bit” at the end of treatment. No patients reported “quite a bit” of interference from urinary frequency at 3 months after radiotherapy, and only 1 patient reported such interference from urinary urgency. Converted values showed comparable results.

“Moderate” (26% vs. 6%) and “severe” (10% vs. 0%) pain or burning with urination increased at the end of treatment, correlating with increases in grade 2 and grade 3 adverse events. These symptoms recovered 3 months after treatment (4% with “moderate” and 0% with "severe”). Urinary incontinence was uncommon.

Finally, erectile function steadily declined from baseline to 3 months after radiotherapy. At 3 months after radiotherapy, fewer patients had normal erectile function (27% vs. 58%), and a majority of patients reported “severe” or “very severe” (grade 3) erectile dysfunction (57% vs. 30%), compared to baseline. Of note, all patients received ADT concurrently with radiation.

### Comparison of IR-CTCAE and PRO-CTCAE

The direct comparison of the IR-CTCAE with PRO-CTCAE is shown in Fig. 2 (GI toxicity) and Fig. 3 (GU toxicity). The degree of concordance is explored in Table 3, where the rates of exact match, investigator under-estimates, and investigator over-estimates are shown in comparison with PRO-CTCAE. Uncommon adverse events (fecal incontinence, urinary incontinence) were not included. Proctitis in IR-CTCAE was excluded because of its imperfect correlation with abdominal pain in PRO-CTCAE (Table 1).

**Table 3:** Correlations between investigator-reported outcomes and PROs are considered. EOT stands for end of treatment; 3 months refers to the time point 3 months after the completion of radiotherapy; and Q1 and Q2 refer to questions 1 and 2 for a specific toxicity (question 2 addresses the degree to which symptoms interfere with a patient’s quality of life).

Adverse event	Exact match	Investigator underestimates	Investigator overestimates	Discordance by ≥ 2 grades	Kappa statistic
**Diarrhea** Baseline EOT 3 months	45/53 (85%) 31/47 (66%) 39/47 (83%)	8/55 (15%) 13/47 (28%) 7/47 (15%)	0/55 (0%) 3/47 (6%) 1/47 (2%)	2/55 (4%) 5/47 (11%) 2/47 (4%)	0.33 (0.04–0.63) 0.32 (0.12–0.52) 0.27 (-0.01–0.54)
**Urinary frequency** Q1: baseline Q1: EOT Q1: 3 months Q2: baseline Q2: EOT Q2: 3 months	23/53 (43%) 21/49 (43%) 22/48 (46%) 17/52 (33%) 9/49 (18%) 7/48 (15%)	11/53 (21%) 17/49 (35%) 8/48 (17%) 0/52 (0%) 1/49 (2%) 0/48 (0%)	19/53 (36%) 11/49 (22%) 18/48 (38%) 35/52 (67%) 39/49 (80%) 41/48 (85%)	5/53 (9%) 2/49 (4%) 2/48 (4%) 5/52 (10%) 9/49 (18%) 4/48 (8%)	0.16 (0.02–0.31) 0.07 (−0.13–0.26) 0.18 (0.03–0.33) 0.06 (0.00–0.33) 0.06 (−0.01–0.13) − 0.03 (−0.09–0.03)
**Urinary urgency** Q1: baseline Q1: EOT Q1: 3 months Q2: baseline Q2: EOT Q2: 3 months	33/54 (61%) 24/48 (50%) 29/47 (62%) 27/52 (52%) 20/48 (42%) 24/47 (51%)	8/54 (15%)15/48 (31%) 9/47 (19%) 1/52 (2%) 4/48 (8%) 2/47 (4%)	13/54 (24%) 9/48 (19%) 9/47 (19%) 24/52 (46%) 24/48 (50%) 21/47 (45%)	5/54 (9%) 2/48 (4%) 0/47 (0%) 3/52 (6%) 1/48 (2%) 0/47 (0%)	0.29 (0.09–0.49) 0.15 (-0.05–0.34) 0.30 (0.07–0.54) 0.04 (-0.08–0.17) 0.11 (-0.03–0.25) 0.13 (-0.02–0.29)
**Urinary tract pain** Baseline EOT 3 months	43/53 (81%) 25/47 (53%) 38/47 (81%)	7/53 (13%) 21/47 (45%) 9/47 (19%)	3/53 (6%) 1/47 (2%) 0/47 (0%)	2/53 (4%) 8/47 (17%) 1/47 (2%)	0.24 (−0.05–0.55) 0.26 (0.12–0.39) 0.07 (−0.05–0.19)
**Erectile dysfunction** Baseline EOT 3 months	9/28 (32%) 5/21 (24%) 2/27 (7%)	10/28 (36%) 9/21 (43%) 20/27 (74%)	9/28 (32%) 7/21 (33%) 5/27 (19%)	10/28 (36%) 8/21 (38%) 15/27 (56%)	0.18 (−0.06–0.43) − 0.04 (−0.27–0.18) − 0.04 (−0.16–0.08)

Investigator-reported diarrhea had relatively good concordance with patient-reported diarrhea at baseline and 3 months after radiotherapy, but there was still a trend towards underestimation in both cases (15% in each time point). The underestimation was worse at the end of treatment, with a 28% rate of underestimation and an 11% rate of discordance by ≥ 2 toxicity grades (all of which were underestimates). Urinary tract pain demonstrated a similar overall pattern. Concordance was high at baseline (over 80% of cases). At EOT, however, only 53% of investigator-reported pain matched patient-reported pain, with a 45% rate of underestimation and a 17% rate of discordance by ≥ 2 toxicity grades (all of which were underestimates). The Kappa statistic generally demonstrated fair concordance for these adverse events.

Urinary urgency and frequency were both considered through two questions. In these domains, IR-CTCAE matched exactly with PRO-CTCAE in only 15 – 62% of cases across all time points. The frequency of symptoms (question 1) tended be overestimated prior to treatment (36% and 24% for urinary frequency and urgency, respectively), but they were generally underestimated at the end of treatment (35% and 31% for urinary frequency and urgency, respectively). The Kappa statistic also fell significantly for both of these adverse events from a slight to fair concordance to no concordance at the end of treatment. The degree of interference with daily activities from these symptoms (question 2) was consistently overestimated by investigators (45% − 85%) across all time points. Poor concordance between IR-CTCAE and PRO-CTCAE for the questions concerning interference with daily activities was noted with low Kappa statistic (Kappa: −0.03–0.13).

Finally, concordance between IR-CTCAE and PRO-CTCAE was particularly poor for erectile dysfunction. The rates of discordance by ≥ 2 toxicity grades (36% − 56%) were actually higher than the rates of exact match (7% − 32%). Investigator underestimation was most common (36% − 74%), but overestimation also occurred (19% − 33%). This poor concordance is reflected by a low Kappa statistic (Kappa: −0.04–0.18).

## Discussion

We present a detailed analysis comparing IR-CTCAE to PRO-CTCAE, and multiple important differences were noted. Overall, there was low agreement between IR-CTCAE and PRO-CTCAE, and investigators tended to underestimate the frequency and severity of urinary symptoms and diarrhea at the end of treatment. Even so, our findings suggest that IR-CTCAE and PRO-CTCAE are complementary, and consideration of both tools may allow for a more complete understanding of patient side effect burden and its impact on quality-of-life [21]. While IR-CTCAE is a useful endpoint, the differences identified via PRO-CTCAE suggest the presence of additional information that can be analyzed through the use of both assessments. Finally, our findings point towards an overall favorable toxicity profile for high-risk or unfavorable intermediate-risk prostate cancer treated with proton beam therapy [22].

There is a dearth of validated tools and literature to correlate patient-reported outcomes with investigator-reported outcomes [23], [24]. Such studies generally reveal low agreement between patient- and investigator-reported outcomes, with investigators often underreporting symptom severity [18], [19]. Other studies have found that clinicians are best able to assess patient toxicities with more severe side effects [25]. In our study, a novel correlation between PRO-CTCAE and IR-CTCAE for many important GI and GU adverse events was developed to examine the concordance between the two assessment tools. Correlating PRO-CTCAE with IR-CTCAE involved both the non-converted, raw data, of PRO-CTCAE and the converted grade of PRO-CTCAE (by equating a descriptive term for each PRO-CTCAE raw data to that of the IR-CTCAE grading score, Table 1). The degree of concordance between PRO-CTCAE and IR-CTCAE was similar with either using the non-converted, raw data, of PRO-CTCAE or using the converted grade of PRO-CTCAE. This result suggests that our correlation effort yielded an appropriate comparison between the two instruments for those GI and GU domains examined in this study. We strongly encourage further study and validation of this new method for correlating PRO-CTCAE with IR-CTCAE.

PROs have shown the potential to guide treatment decisions, and their use has increased in recent clinical trials [17], [26]. Our results indicate that urinary symptoms were underestimated by investigators compared to PRO-CTCAE in about one-third of cases. This high rate of discordance at the end of treatment suggests that this time period represents an important phase of treatment during which a clinician needs to carefully assess for side effects of radiotherapy [27]. These results suggest that patients may not always volunteer this information at the last management visit. Instead, it may be that direct questioning regarding the presence or absence of these symptoms (e.g. via PRO-CTCAE) is required, and we encourage investigators to directly ask patients regarding the development of these side effects. It is also noteworthy that rates of symptom overestimation by investigators were high at baseline, potentially clouding accurate assessment of changes in those symptoms throughout the treatment course. Medications can be prescribed to address urinary frequency and urgency, and our results suggest that additional intervention may prove beneficial.

Significant differences were also noted regarding the interference of urinary symptoms with daily activities. Patients reported low rates of interference while investigators overestimated the impact of urinary symptoms on daily activities. It may be that interference with daily activities is too distinct a question to directly correlate with CTCAE. Even so, the striking differences between PRO-CTCAE and IR-CTCAE in this setting suggest that this area is inadequately addressed by our current practices of toxicity reporting. Ideally, investigators would seek to capture both the frequency and degree of interference with quality of life caused by a particular side effect. Current investigator-report outcomes likely inadequately address this aspect of treatment toxicity.

An analysis of over 15,000 patients in the Danish Prostate Cancer Registry suggests that overall quality-of-life was most adversely affected by sexual function, regardless of treatment modality [28]. Furthermore, another study found that regret about the treatment choice was more common among those who experienced more treatment-related symptoms during the year after treatment [29]. Though the reason for the decline in erectile function is likely multi-factorial (e.g. increasing age, ADT, and radiotherapy), the striking differences between IR-CTCAE and PRO-CTCAE regarding erectile dysfunction in our study suggests a need for close evaluation and frank conversations between patients and healthcare providers.

Limitations of this study chiefly relate to a relatively small number of patients analyzed at each time point. Since only 55 patients answered PRO assessments, more rigorous statistical analysis was not performed. Additionally, it is likely that the correlations drawn between PRO-CTCAE and IR-CTCAE were imperfect and may have influenced our results. Even so, results were quite similar between the converted and non-converted PROs, suggesting that any loss through this process was minimal. Finally, proctitis in IR-CTCAE was difficult to address robustly in this analysis because it correlated poorly with ‘abdominal pain’ in PRO-CTCAE. There is no domain in the current PRO-CTCAE that can be correlated well with proctitis in IR-CTCAE. Future studies should aim to more completely address this adverse event.

## Conclusions

Our study shows low agreement between IR-CTCAE and PRO-CTCAE in the setting of proton therapy for prostate cancer. Compared to patient-reported outcomes, physicians underestimated the frequency and severity of urinary symptoms and diarrhea at the end of treatment. Continued use of PROs should be strongly encouraged.

## Declaration of Competing Interest

The wife of Dr. Kowalchuk is a senior technical product manager for GE Healthcare. No other authors have relevant conflicts of interest to report.

## References

[1] Siddiqui F., Liu A.K., Watkins-Bruner D., Movsas B. (2014). Patient-reported outcomes and survivorship in radiation oncology: overcoming the cons. J Clin Oncol.

[2] Watkins Bruner D., Bryan C.J., Aaronson N., Blackmore C.C., Brundage M., Cella D. (2007). Issues and challenges with integrating patient-reported outcomes in clinical trials supported by the national cancer institute–sponsored clinical trials networks. J Clin Oncol.

[3] Hauth F., Bizu V., App R., Lautenbacher H., Tenev A., Bitzer M. (2019). Electronic patient-reported outcome measures in radiation oncology: Initial experience after workflow implementation. JMIR mHealth uHealth.

[4] Amdal C.D., Jacobsen A.-B., Guren M.G., Bjordal K. (2013). Patient-reported outcomes evaluating palliative radiotherapy and chemotherapy in patients with oesophageal cancer: a systematic review. Acta Oncol.

[5] Chow S., Ding K., Wan B.A., Brundage M., Meyer R.M., Nabid A. (2018). Patient reported outcomes after radiation therapy for bone metastases as a function of age: a secondary analysis of the ncic ctg sc—twenty-three randomized trial. Am J Hospice Palliative Med.

[6] Conway J.L., Yurkowski E., Glazier J., Gentles Q., Walter A., Bowering G. (2016). Comparison of patient-reported outcomes with single versus multiple fraction palliative radiotherapy for bone metastasis in a population-based cohort. Radiother Oncol.

[7] Alberts L., Wolff H.B., Kastelijn E.A., Lagerwaard F.J., Hofman F.N., Sharouni S.Y.E. (2019). Patient-reported outcomes after the treatment of early stage non–small-cell lung cancer with stereotactic body radiotherapy compared with surgery. Clin Lung Cancer.

[8] Rao A.D., Sugar E.A., Chang D.T., Goodman K.A., Hacker-Prietz A., Rosati L.M. (2016). Patient-reported outcomes of a multicenter phase 2 study investigating gemcitabine and stereotactic body radiation therapy in locally advanced pancreatic cancer. Pract Radiat Oncol.

[9] Hayakawa T., Kawakami S., Soda I., Kainuma T., Nozawa M., Sekiguchi A. (2019). Dosimetric factors associated with long-term patient-reported outcomes after definitive radiotherapy of patients with head and neck cancer. Radiat Oncol.

[10] Holländer-Mieritz C., Johansen J., Johansen C., Vogelius I.R., Kristensen C.A., Pappot H. (2019). Comparing the patients’ subjective experiences of acute side effects during radiotherapy for head and neck cancer with four different patient-reported outcomes questionnaires. Acta Oncol.

[11] Korzeniowski M., Kalyvas M., Mahmud A., Shenfield C., Tong C., Zaza K. (2016). Piloting prostate cancer patient-reported outcomesin clinical practice. Support Care Cancer.

[12] Reeve B.B., Mitchell S.A., Dueck A.C. (2014). Recommended patient-reported core set of symptoms to measure in adult cancer treatment trials. JNCI: J Natl Cancer Inst.

[13] Donovan J.L., Hamdy F.C., Lane J.A., Mason M., Metcalfe C., Walsh E. (2016). Patient-reported outcomes after monitoring, surgery, or radiotherapy for prostate cancer. N Engl J Med.

[14] Basch E., Dueck A.C., Rogak L.J., Minasian L.M., Kelly W.K., O’Mara A.M. (2017). Feasibility assessment of patient reporting of symptomatic adverse events in multicenter cancer clinical trials. JAMAOncol.

[15] Basch E, Reeve BB, Mitchell SA, et al. Development of the national cancer institute’s patient-reported outcomes version of the common terminology criteria for adverse events (pro-ctcae). J Natl Cancer Inst 2014;106:dju244. 10.1093/jnci/dju244 PMC4200059 25265940

[16] Dueck A.C., Mendoza T.R., Mitchell S.A., Reeve B.B., Castro K.M., Rogak L.J. (2015). Validity and reliability of the us national cancer institute’s patient-reported outcomes version of the common terminology criteria for adverse events (pro-ctcae). JAMAOncol.

[17] Gounder M.M., Mahoney M.R., Van Tine B.A., Ravi V., Attia S., Deshpande H.A. (2018). Sorafenib for advanced and refractory desmoid tumors. N Engl J Med.

[18] Nyrop K.A., Deal A.M., Reeve B.B., Basch E., Chen Y.T., Park J.H. (2020). Congruence of patient-and clinician-reported toxicity in women receiving chemotherapy for early breast cancer. Cancer.

[19] Rammant E., Ost P., Swimberghe M. (2019). Patient-versus physician-reported outcomes in prostate cancer patients receiving hypofractionated radiotherapy within a randomized controlled trial. Strahlenther Onkol.

[20] Basch E, Becker C, Rogak LJ, et al. Composite grading algorithm for the national cancer institute’s patient-reported outcomes version of the common terminology criteria for adverse events (pro-ctcae). Clinical Trials 2020:1740774520975120. 10.1177/1740774520975120 PMC7878323 33258687

[21] Niska J.R., Thorpe C.S., Halyard M.Y., Tan A.D., Atherton P.J., Dueck A.C. (2020). Patient-reported quality-of-life outcomes in relation to provider-assessed adverse events during head and neck radiotherapy. J Patient-Rep Outcomes.

[22] Verma V, Simone CB, Mishra MV. Quality of life and patient-reported outcomes following proton radiation therapy: a systematic review. JNCI: J Natl Cancer Inst 2018;110:341-353. 10.1093/jnci/djx208 29028221

[23] Campbell J.M., O'Callaghan M.E., Raymond E., Vincent A.D., Beckmann K.R., Roder D. (2017). Tools for predicting clinical and patient-reported outcomes in prostate cancer patients undergoing androgen deprivation therapy: a systematic review of prognostic accuracy and validity. Clin Genitourinary Cancer.

[24] O'Callaghan M.E., Raymond E., Campbell J.M. (2017). Patient-reported outcomes after radiation therapy in men with prostate cancer: a systematic review of prognostic tool accuracy and validity. Int J Radiat Oncol Biol Phys.

[25] Wilkie J.R., Mierzwa M.L., Yao J., Eisbruch A., Feng M., Weyburne G. (2019). Big data analysis of associations between patient reported outcomes, observer reported toxicities, and overall quality of life in head and neck cancer patients treated with radiation therapy. Radiother Oncol.

[26] Dueck A.C., Scher H.I., Bennett A.V., Mazza G.L., Thanarajasingam G., Schwab G. (2020). Assessment of adverse events from the patient perspective in a phase 3 metastatic castration-resistant prostate cancer clinical trial. JAMAOncol.

[27] Morgans A.K., Stockler M.R. (2019). Patient-reported outcomes in metastatic castration-sensitive prostate cancer in the adjuvant setting. Eur Urol Focus.

[28] Nguyen-Nielsen M., Møller H., Tjønneland A., Borre M. (2020). Patient-reported outcome measures after treatment for prostate cancer: results from the danish prostate cancer registry (daprocadata). CancerEpidemiol.

[29] van Stam M.-A., Aaronson N.K., Bosch J.L.H.R., Kieffer J.M., van der Voort van Zyp J.R.N., Tillier C.N. (2020). Patient-reported outcomes following treatment of localised prostate cancer and their association with regret about treatment choices. Eur Urol Oncol.

