# Supplementary material for: Assessing concordance between patient-reported and investigator-reported CTCAE after proton beam therapy for prostate cancer
Source: Clin Transl Radiat Oncol. 2021 Sep 15;31:34–41. doi: 10.1016/j.ctro.2021.09.003 (PMC8463742; doi:10.1016/j.ctro.2021.09.003)
Supplement: Supplementary data 1 [file mmc1.docx]

**Supplemental methods**

**Inclusion and exclusion criteria**

Inclusion criteria included patients with biopsy-proven prostate adenocarcinoma and at least one of the following features: Gleason score ≥ 8, clinical stage T3 or T4, prostate specific antigen (PSA) < 100 but > 20 ng/mL. Patients were also eligible if they presented with unfavorable intermediate-risk prostate cancer, defined as T1 or T2, Gleason score 4 + 3, and PSA 10-20 ng/mL. Exclusion criteria included patients diagnosed with distant or pelvic lymph node metastases after staging work-up consisting of bone scan and imaging of the abdomen and pelvis (CT or MRI).

**CT simulation**

At least 1 day prior to CT simulation, four carbon makers were implanted into the prostate gland through a trans-perineal or trans-rectal approach using trans-rectal ultrasound guidance, to assist with daily image guidance. Simulation was performance with a full bladder, and patients attempted to have a bowel movement in the morning before simulation, as well as 60 minutes prior to simulation, to minimize residual stool or bowel gas. Patients were placed in the supine position, and a custom vacuum-lock bag and an indexed knee cushion were utilized to immobilize the lower extremities. CT scans had a 2 mm slice thickness and covered the iliac crests to the mid-femur. MRI of the pelvis was also obtained using the same patient positioning.

**Volume guidelines**

CT and MRI images were co-registered, with the intra-prostatic carbon markers used as references. The Eclipse treatment planning system (Varian Medical Systems, Palo Alto, CA) was used for all patients. Two clinical target volumes (CTVs) were delineated. The high-dose CTV included the prostate and seminal vesicles, which were treated to 67.5 Gy in 2.7 Gy fractions over five weeks. The low-dose CTV included the pelvic lymph nodes, which were treated to 45 Gy in 1.8 Gy fractions over the same five-week timeframe. Assuming an α/β ratio of 1.5 to 3 for prostate cancer, this regimen delivers an equivalent of 80.2 – 85.9 Gy in 1.8 fractions to the high-dose CTV.

CTV delineation was chiefly performed using CT images, and MRI images primarily aided with the high-dose CTV volume refinement. The Radiation Therapy Oncology Group (RTOG) consensus guidelines were used to define CTVs (1). The extent of the seminal vesicles included within the high-dose CTV was left at the discretion of the treating physician’s interpretation of the clinical and pathologic disease features. The low-dose CTV included the obturator, external iliac, proximal internal iliac, distal common iliac, and presacral nodes. Distal common iliac nodes were defined as up to the level of the sacral promontory or the lumbosacral junction, and presacral nodes extended inferiorly to the level of S3. A 7 mm margin in 3 dimensions was used for the iliac vessels, and a 10mm margin was used for the presacral nodes from the anterior sacral bone. Pelvic organs at risk (OARs) were contoured according to the RTOG guidelines. These included the large bowel, small bowel, rectum, bladder, femoral heads, and penile. OARs, pelvic musculature, and bones were cropped out of the low-dose CTV. The high-dose planning target volume (PTV) was obtained with a 5 mm expansion from the high-dose CTV (except for a 4 mm expansion posteriorly), and the low-dose PTV was obtained with a 4-5 mm expansion of the low-dose CTV.

**Treatment planning**

Intensity modulated proton therapy (IMPT) was determined through the use of pre-defined dose-volume histogram (DVH) objectives for targets volumes and OARs. Key metrics assessed for target volumes included the dose received by at least 98% of the volume (D98%) and D2%, as well as the volume receiving at least 100% of the prescribed dose (V100%) and V107%. OARs were analyzed with mean dose, maximum dose (D2cc), as well as the following objectives specific to particular OARs: (1) bladder: D2cc < 72.9 Gy, V66Gy < 8%, V61Gy < 11%, V 57 Gy < 15%, and V36 Gy < 33%; (2) rectum: D2cc < 71.5 Gy, V66Gy < 9%, V61Gy < 12%, V 57 Gy < 15%, V53 Gy < 17%, and V44 Gy < 24%; and (3) small bowel: max < 52 Gy, V50Gy < 2 cc, V45 Gy < 150 cc, and V30 Gy < 300 cc.

Optimized proton beam therapy plans were generated through the Eclipse treatment planning system. Inverse optimization was used for dose distributions from pencil beam spots scanned for a cloud covering targets and OARs. Modulation of each beam spot and gantry angle selection were individualized for each patient’s anatomy and target volumes. Most commonly, two opposed lateral beams were used, with spot spacing set to 3 mm.

**Radiotherapy delivery**

Prior to treatment delivery, daily matching of the prostate position was performed with onboard orthogonal-kV imaging of the intra-prostatic carbon markers. A verification CT scan was obtained weekly to account for uncertainty of pelvic lymph node position. Intra-prostatic carbon markers and pelvic bones were used for matching of the verification and planning CT scans to allow for assessment of CTV coverage, relative to the CT verification scan. New IMPT plans were generated when CTV coverage was inadequate.

Of note, patients also received androgen-deprivation therapy (ADT) for 4-36 months as part of the treatment paradigm of high or unfavorable intermediate risk prostate cancer. ADT was initiated 2 months prior to radiotherapy and consisted of a LHRH agonist (goserelin or leuprolide). Bicalutamide was also given for 2-4 weeks at the start of ADT (50 mg, po, once daily).

**PRO-CTCAE and IR-CTCAE correlation**

Two physicians independently developed this correlation upon careful assessment of the PRO-CTCAE and IR-CTCAE. This effort involved equating the descriptive term of each PRO-CTCAE to its comparable IR-CTCAE grading score. This correlation was performed for each PRO-CTCAE question, and each descriptive term of PRO-CTCAE was individually assessed and assigned with its comparable IR-CTCAE grading score (shown in Table 1). Both physicians then discussed their independently developed correlation results, and negligible disagreement was found. In this manner, a group consensus of how to correlate between the descriptive term of each PRO-CTCAE and its comparable IR-CTCAE grading score was obtained.

**REFERENCES**

1. Lawton CA, Michalski J, El-Naqa I, et al. Rtog gu radiation oncology specialists reach consensus on pelvic lymph node volumes for high-risk prostate cancer. *International Journal of Radiation Oncology* Biology* Physics* 2009;74:383-387.
